# Facile Synthesis of Ternary g-C_3_N_4_@BiOCl/Bi_12_O_17_Cl_2_ Composites With Excellent Visible Light Photocatalytic Activity for NO Removal

**DOI:** 10.3389/fchem.2019.00231

**Published:** 2019-04-11

**Authors:** Wendong Zhang, Yi Liang

**Affiliations:** Chongqing Key Laboratory of Inorganic Functional Materials, Department of Scientific Research Management, Chongqing Normal University, Chongqing, China

**Keywords:** facile synthesis, g-C_3_N_4_@BiOCl/Bi_12_O_17_Cl_2_, visible light, photocatalytic activity, nitrogen oxide (NO) removal

## Abstract

In this study, novel two-dimensional (2D) g-C_3_N_4_@BiOCl/Bi_12_O_17_Cl_2_ composites have been fabricated through a facile deposition-precipitation process. The as-prepared photocatalysts were characterized by XRD, SEM, TEM, XPS, UV-vis DRS, PL, Photocurrent, EIS, ESR, and N_2_ adsorption-desorption. The photocatalytic activities were investigated through NO removal test in gas under visible light irradiation (λ > 420 nm). The g-C_3_N_4_@BiOCl/Bi_12_O_17_Cl_2_ composites exhibit enhanced visible light absorption and photo-induced electron-hole separation efficiency, compared with pristine g-C_3_N_4_ and BiOCl/Bi_12_O_17_Cl_2_. The intimated contact interfaces between g-C_3_N_4_ and BiOCl/Bi_12_O_17_Cl_2_ nanosheets are responsible for the more efficient photochemical interactions. The present work provides a new direction to develop a class of ternary g-C_3_N_4_-based visible-light-driven photocatalysts for environmental purification.

## Introduction

In the past decades, with the rapid development of modern industrial society, a large amount of highly harmful and toxic contaminants have been discharged into environmental system, which have been the focus of world attention (Han et al., 2017; Li et al., 2018). It remains a great challenge to completely achieve the degradation of environmental contaminants through the conventional treatment process, especially for the low-level concentrations of contaminants (Jiang et al., 2017, 2018; Zhong et al., 2017).

Photocatalysis, as a novel technique, have potential application in degradation low-level concentrations of environmental contaminants under visible-light irradiation (Xiong et al., 2015; Zheng and Zhang, 2016; Guan et al., 2017; Jin et al., 2018). Up to now, although a large number of photocatalysts have been explored for environmental purification, most of them still suffer from the limited utilization of solar light, resulting in quite low visible light photocatalytic activity (Chibac et al., 2017; Zhang et al., 2017). Hence, it is desirable to develop highly efficient visible-light-driven photocatalysts for satisfying the requirements of practical applications.

Currently, graphitic carbon nitride (g-C_3_N_4_), an organic semiconductor photocatalyst, has attracted intensive research interest in energy conversion and environmental remediation fields, largely due to its typical physicochemical properties, such as suitable band gap, good chemical and thermal stability, environmental friendly, etc (Wang et al., 2011; Dong and Zhang, 2013; Dong et al., 2015). However, the photocatalytic efficiency of g-C_3_N_4_ is far below the requirement of practical applications, mainly due to its fast recombination of photo-excited electron-hole pairs. So far, various strategies have been developed to improve the photocatalytic performance of g-C_3_N_4_, including electronic structure engineering, nanostructure optimization, and heterojunction construction (Cao et al., 2015; Zhao et al., 2015; Ong et al., 2016). Furthermore, it is well-known that the g-C_3_N_4_ nanosheets can provide a good two-dimensional surface and interface platform for growth of other nanostructured semiconductors. In particular, the heterojunction with other semiconductor photocatalysts has been regarded as an attractive and effective solution in enabling the efficient separation of photo-excited electron-hole pairs, which not only helps to prolong the life-time of photo-excited charge carriers, but also endows g-C_3_N_4_-based heterojunctions with more abundant active sites, leading to the significantly enhancement of photocatalytic performance. Successful examples include g-C_3_N_4_/TiO_2_ (Wei et al., 2016), g-C_3_N_4_/MoS_2_ (Hou et al., 2013), g-C_3_N_4_/BiOBr (Sun et al., 2014), g-C_3_N_4_/WO_3_ (Huang et al., 2013), g-C_3_N_4_/CdS (Liu, 2015), g-C_3_N_4_/g-C_3_N_4_ (Dong et al., 2013), g-C_3_N_4_/(BiO)_2_CO_3_ (Zhang et al., 2014), g-C_3_N_4_/graphene (Kim et al., 2016), and g-C_3_N_4_/Ag (Olga et al., 2016), etc. However, little information about ternary g-C_3_N_4_@BiOCl/Bi_12_O_17_Cl_2_ heterojunction for visible light photocatalytic removal of NO has been reported.

Here, we report a highly cost-effective method based on the *in situ* self-assembly of BiOCl/Bi_12_O_17_Cl_2_ binary nanoplates onto the surface of g-C_3_N_4_ nanosheets at room temperature. It takes into consideration advantages of well-matched band structures among g-C_3_N_4_, BiOCl and Bi_12_O_17_Cl_2_, the as-prepared ternary g-C_3_N_4_@BiOCl/Bi_12_O_17_Cl_2_ heterojunctions exhibit apparent characteristics including larger surface area, improved visible light absorption ability, and efficient separation of photo-induced charge carries, which are extremely favorable for improving the photocatalytic activity.

### Fabrication of g-C_3_N_4_@BiOCl/Bi_12_O_17_Cl_2_ Composites

The pure g-C_3_N_4_ nanosheets and TiO_2_ powders were fabricated according to the previous reports (Dong et al., 2011; Zhang et al., 2014), respectively. It's a typical synthesize that 1.33 g of BiCl_3_ and 0.25 g of as-obtained g-C_3_N_4_ were added to 50 mL absolute ethyl alcohol and then were ultrasonicated for 30 min. Afterwards, dripping 12.6 mL of NaOH solution (2.0 mol/L) dropwise into BiCl_3_ solution and then stirring vigorously for 4 h at room temperature. After that, filtering and washing the resulting precipitate for times with distilled water and ethanol. The final samples were obtained after drying under vacuum at 60°C for 24 h. Five types of samples were prepared. The mass rations of BiOCl/Bi_12_O_17_Cl_2_ to g-C_3_N_4_ are 1:2, 1:4, 1:1, 2:1, and 4:1, respectively. Accordingly, the final samples were labeled as BOC-CN-1-2, BOC-CN-1-4, BOC-CN-1-1, BOC-CN-2-1, and BOC-CN-4-1, respectively. The pure BiOCl/Bi_12_O_17_Cl_2_ samples were synthesized under the same conditions without adding g-C_3_N_4_ nanosheets, and the g-C_3_N_4_@BiOCl/Bi_12_O_17_Cl_2_ sample was labeled as BOC.

### Materials Characterization

In a typical analysis of the crystal phase of the as-obtained samples, X-ray diffraction with Cu Kα radiation (XRD: model D/max RA, Japan) was applied. Meanwhile scanning electron microscope (SEM, JEOL model JSM-6490, Japan) and transmission electron microscopy (TEM: JEM-2010, Japan) were utilized to characterize the morphology and structure. And the surface properties were examined by X-ray photoelectron spectroscopy with Al Kα X-rays (hν = 1486.6 eV) radiation operated at 150 W (XPS: Thermo ESCALAB 250, USA). By using a Scan UV-vis spectrophotometer (UV-vis DRS: UV-2450, Shimadzu, Japan) equipped with an integrating sphere assembly and BaSO_4_ as reflectance sample, the UV-vis diffuse reflection spectra was gained. Nitrogen adsorption-desorption was conducted on a nitrogen adsorption apparatus (ASAP 2020, USA) to insure the specific surface areas and total pore volumes. Photoluminescence (PL: F-7000, HITACHI, Japan) was used to investigate the charge transfer properties. ESR spectrometer (FLsp920, England) was applied to detect the electron spin resonance (ESR) signals of •OH and ${•\text{O}}_{2}^{-}$, respectively. The photocurrent measurements (CHI 660B electrochemical system: Shanghai, China) and electrochemical impedance spectroscopy (EIS) were carried out to analyze the photo-generated charge separation properties. All the samples were degassed at 150°C prior to measurements.

### Appraisement of Photocatalytic Activity

The photocatalytic activity was investigated by removal of NO at ppb (1 × 10^−9^) levels in a continuous flow reactor at ambient temperature. The volume of the rectangular reactor, made of stainless steel and covered with Saint-Glass, was 4.5 L (30 × 15 × 10 cm). A 100-W commercial tungsten halogen lamp (THL100, Beijing, China) was vertically placed outside the reactor, and the light spectra range (see Supplementary Figure 1) from 350 to 2,400 nm. A UV cut-off filter (Aike UBG-420, Shenzhen, China) was adopted to remove UV light in the light beam. Photocatalyst (0.2 g) was coated onto a dish with a diameter of 12.0 cm, and the irradiance received by the photocatalyst powder is about 0.66 W/cm^2^. The coated dish was then pretreated at 70°C to remove water in the suspension. The catalyst adhesion on the dish was firm enough to avoid the erosion (or removal) of the catalyst during air flowing. The NO gas was acquired from a compressed gas cylinder at a concentration of 100 × 10^−6^ NO (N_2_ balance, BOC gas). The initial concentration of NO was diluted to about 500 × 10^−9^ by the air stream. The desired relative humidity (RH) level of the NO flow was controlled at 50% by passing the zero air streams through a humidification chamber. The gas streams were premixed completely by a gas blender, and the flow rate was controlled at 2.4 L·min^−1^ by a mass flow controller. After the adsorption-desorption equilibrium was achieved, the lamp was turned on. The concentration of NO was continuously measured by a chemiluminescence NO analyzer (Thermo Environmental Instruments Inc., 42i-TL), which monitors NO, NO_2_, and NO_*x*_ (NO_*x*_ represents NO + NO_2_) with a sampling rate of 1.0 L·min^−1^. The removal ratio (η) of NO was calculated by η (%) = (1-*C*/*C*_0_) × 100%, where *C* and *C*_0_ are concentrations of NO in the outlet stream and the feeding stream, respectively.

## Results and Discussion

**Figure 1:**
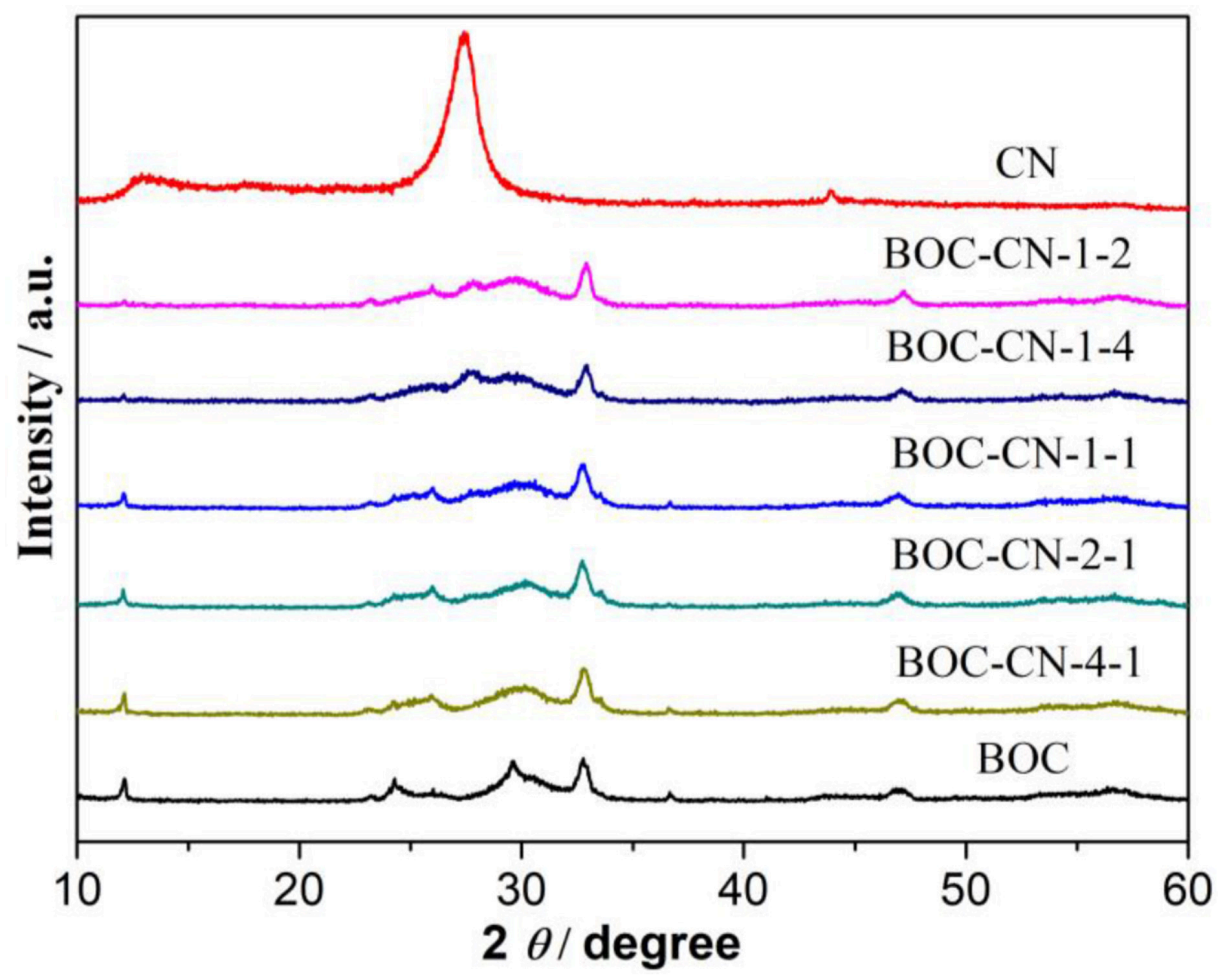
The XRD patterns of the BOC, CN, and BOC-CN samples.

The phase structures of the obtained samples were investigated by XRD patterns. As shown in Figure 1, the BOC sample exhibits typical XRD peaks of BiOCl and Bi_12_O_17_Cl_2_ (He et al., 2016; Huang et al., 2016), thus suggesting the formation of BiOCl/Bi_12_O_17_Cl_2_ heterojunctions, the result is good agreement with the previous reports (Zhang et al., 2017). For the pure CN sample, the typical diffraction peaks appeared at 27.4 and 13.1° are indexed to the g-C_3_N_4_ (002) and (100) planes (Wang et al., 2011; Cao et al., 2015), respectively. Based on further observation, the peak intensity of the CN decreased with increasing BOC content, implying that interactions exist between the BOC and CN. Finally, two peaks of CN cannot be observed in the BOC-CN composites, which can be ascribed to a good dispersion of BOC onto the surface of CN.

**Figure 2:**
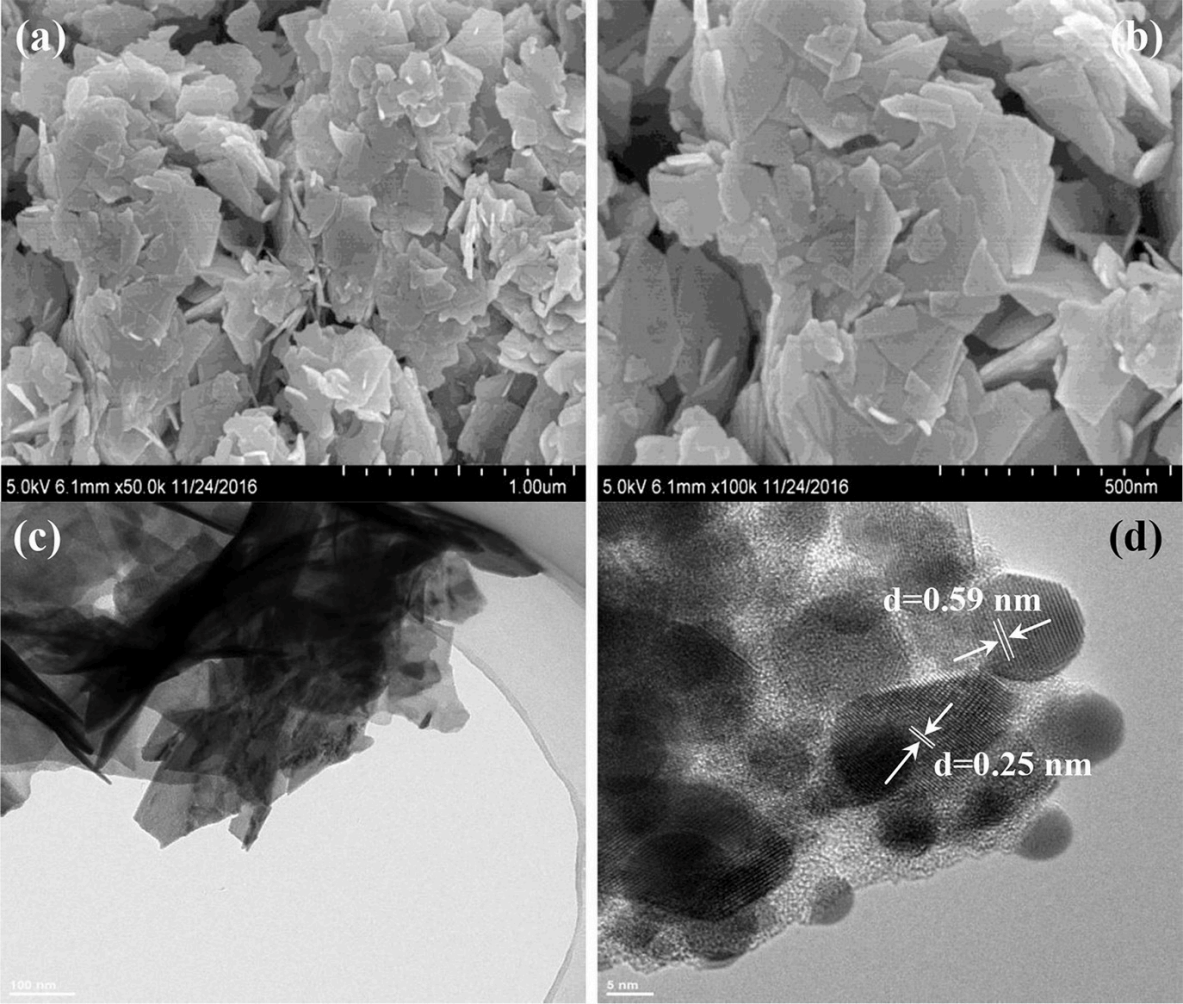
SEM **(a,b)** and TEM **(c,d)** images of BOC samples.

**Figure 3:**
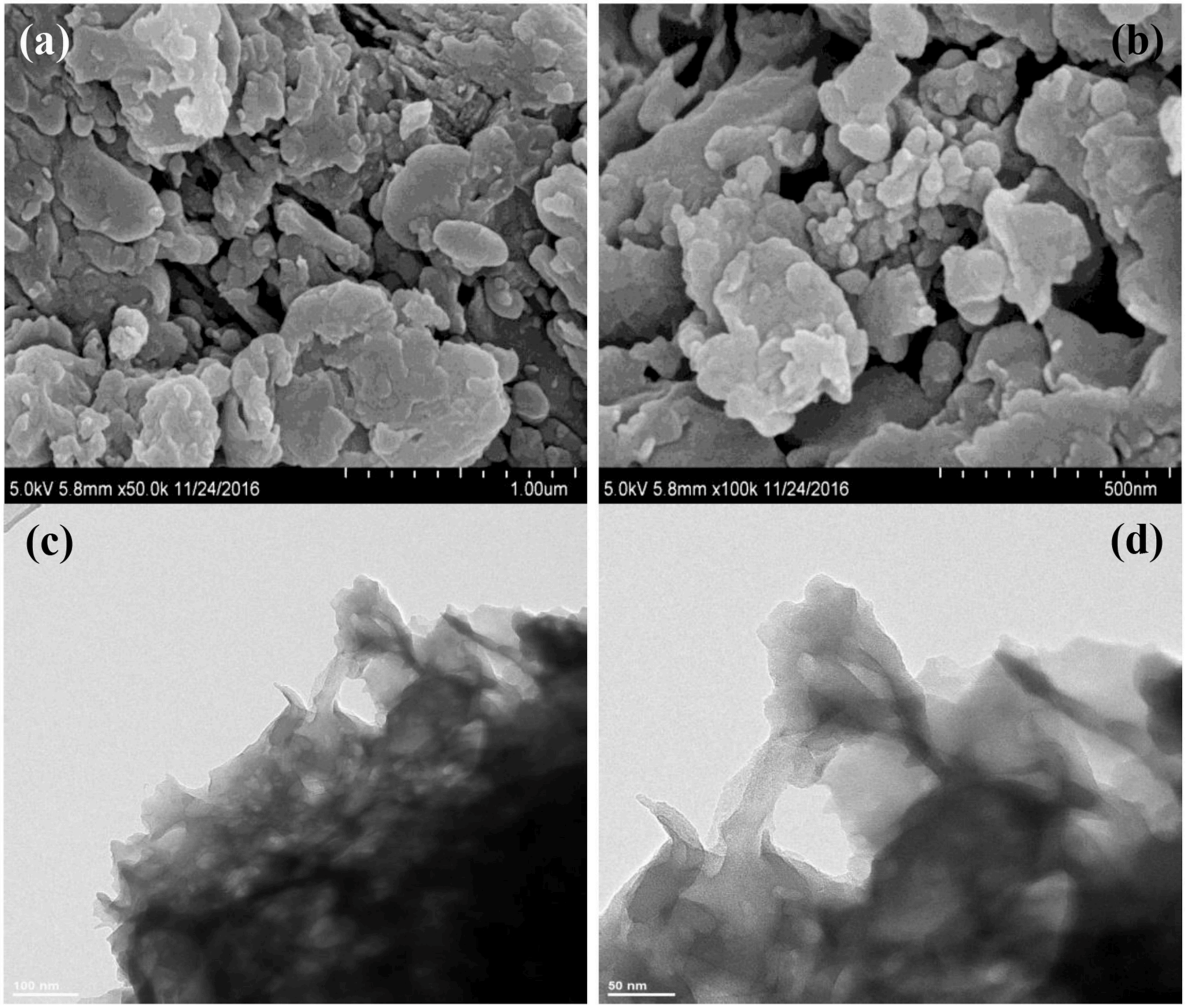
SEM **(a,b)** and TEM **(c,d)** images of CN samples.

**Figure 4:**
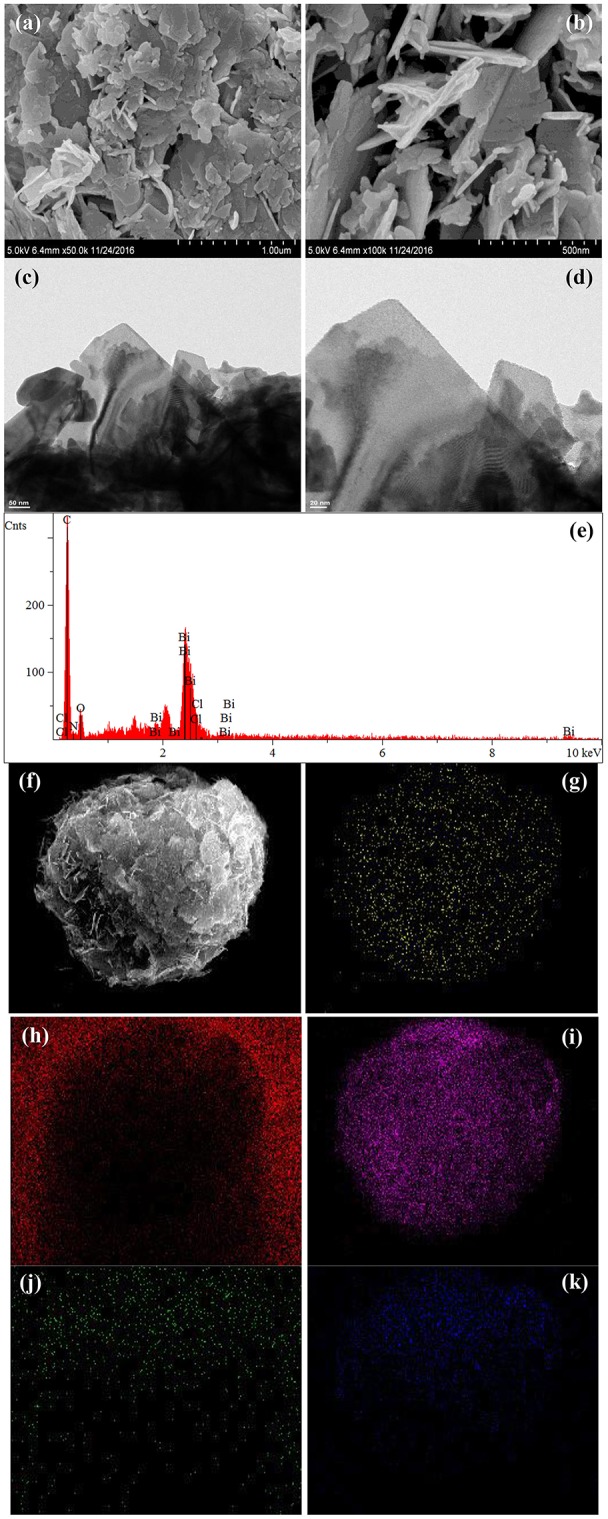
SEM **(a,b)** and TEM **(c,d)** images of BOC-CN. EDS elemental mapping **(e–k)** of the same region, indicating the spatial distribution of Bi **(g)**, C **(h)**, Cl **(i)**, N **(j)**, and O **(k)**, respectively.

The morphologies and microstructures of BOC, CN, and BOC-CN were characterized by SEM and TEM. For BOC samples (Figure 2), the layered and irregular microstructures consist of smooth nanosheets with different sizes. Moreover, there are two different fringes with the lattice spacing of 0.59 and 0.25 nm (Figure 2d), which can be indexed to the (006) crystal plane of Bi_12_O_17_Cl_2_ and (003) crystal plane of BiOCl, respectively. For CN samples (Figure 3), it presents the lamellar morphology is composed of numerous nanosheets with a much looser pore structure. As shown in Figures 4a–d, the BOC-CN composites also consist of a large number of layered nanosheets with different shapes. Obviously, the BOC nanosheets were *in situ* growth on the surface of g-C_3_N_4_, resulting in the formation of closely interface in g-C_3_N_4_@BiOCl/Bi_12_O_17_Cl_2_ composites, which is beneficial for the separation and transfer of the photo-induced electron-hole pairs. The surface element dispersion state of BOC-CN composites are studied by EDS mapping. As shown in Figures 4e–k, the Bi, C, Cl, N, and O elements are uniformly distributed in BOC-CN samples.

**Figure 5:**
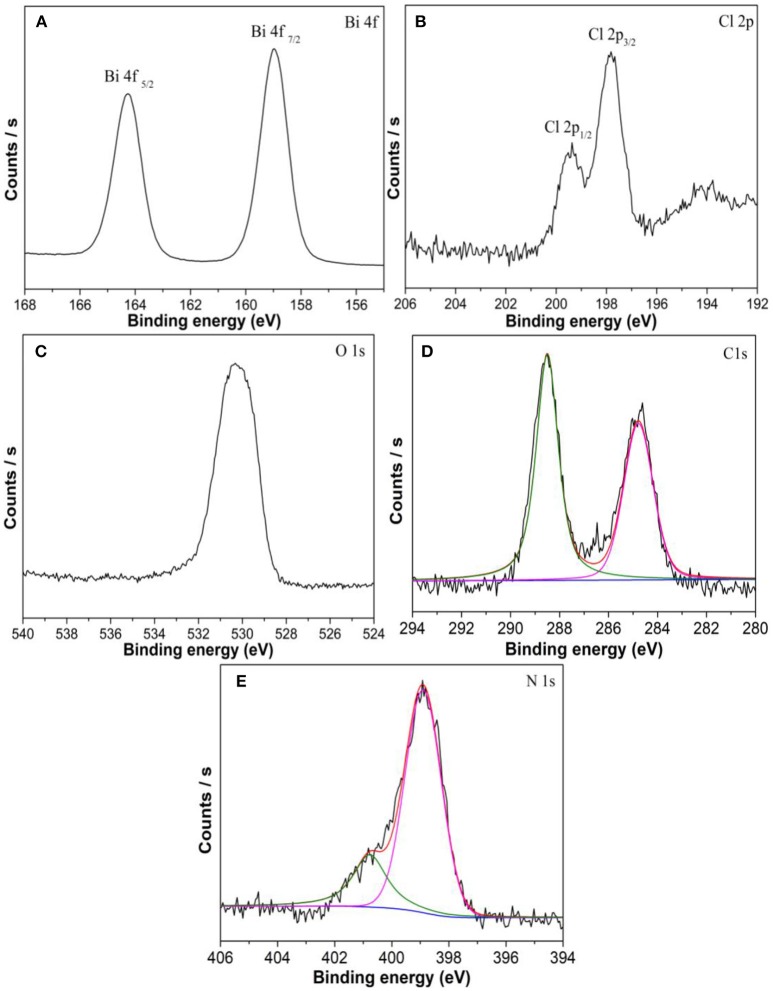
XPS spectra of **(A)** Bi 4f, **(B)** Cl 2p, **(C)** O1s, **(D)** C1s, and **(E)** N1s in BOC-CN.

The XPS measurements were applied to verify the composition and chemical state of the elements. Two peaks at 159.1 and 164.3 eV are consistent with Bi4f_7/2_ and Bi4f_5/2_ (Figure 5A), respectively. The XPS spectrum for Cl shows two peaks at 197.8 and 199.4 eV attributed to Cl2p_3/2_ and Cl2p_1/2_ (Figure 5B), respectively. The peak centered at 530.3 eV that corresponds to the binding energy of O 1s (Figure 5C) (Bi et al., 2016; Zhang et al., 2017). The C peak at 284.8 eV can be ascribed to the adventitious carbon atom. Figure 5D shows one peak at 288.5 is identified as overlapped peaks of N-C = N and the O-C = O. Two different peaks are observed in Figure 5E, the N peak at 398.9 eV correspond to the C = N-C and the N peak at 400.8 eV is attributed to the residual amino groups (Zhang et al., 2014).

**Figure 6:**
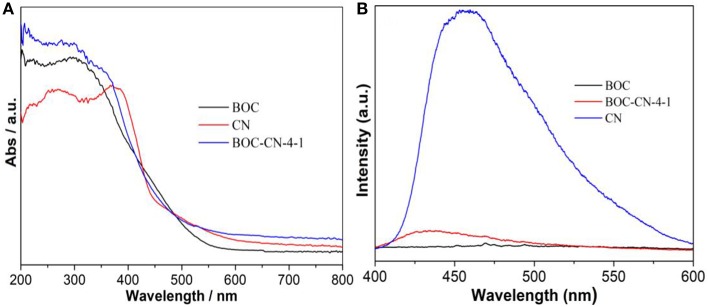
UV-vis diffuse reflectance spectra **(A)** and PL spectra **(B)** for the BOC, CN, and BOC-CN-4-1 samples.

The optical absorption property of the as-obtained samples was investigated by UV-vis DRS. As can be seen from Figure 6A, BOC and CN exhibit absorbance edge around 550 and 475 nm, respectively, displaying that BOC and CN possess good visible light absorption ability. Interestingly, BOC-CN-4-1 shows relatively stronger visible light absorption ability lies in the range of 550–800 nm, because of the synergetic effect between BOC and CN. Figure 6B shows the PL spectra for BOC, CN, and BOC-CN-4-1 using the exciting light of 320 nm. Compared to CN, the PL intensity of BOC-CN-4-1 significantly decreases, demonstrating that the interface interaction between BOC and CN could inhibit the recombination rate of photo-generated electrons and holes.

**Figure 7:**
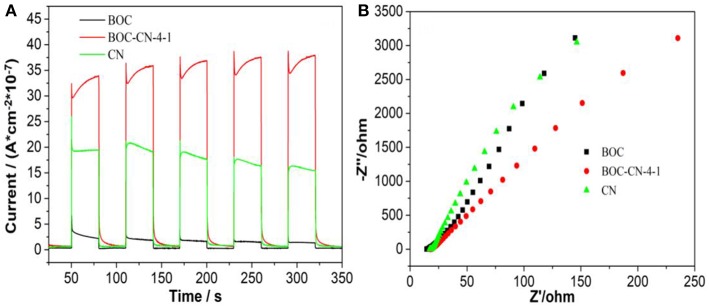
Photocurrent response **(A)** and Nyquist plots **(B)** for BOC, CN, and BOC-CN-4-1 samples under visible light irradiation (λ ≥ 420 nm, [Na_2_SO_4_] = 0.5 M).

The photocurrent and electrochemical impedance experiments were used to investigated the photo-generated charges separation and transfer property of BOC, CN, and BOC-CN-4-1 samples under visible light irradiation (Zhang et al., 2018a). Compared with the pure BOC and CN, it was interesting to find that BOC-CN-4-1 exhibits significantly enhanced photocurrent density (Figure 7A), suggesting that BOC-CN-4-1 possesses higher photo-generated charge separation property. As can be seen from the Figure 7B, the arc radius on the EIS Nyquist plot of BOC-CN-4-1 was smaller than that of the pure BOC and CN, demonstrating that BOC-CN-4-1 has much more efficient photo-generated charge separation and transfer property.

**Figure 8:**
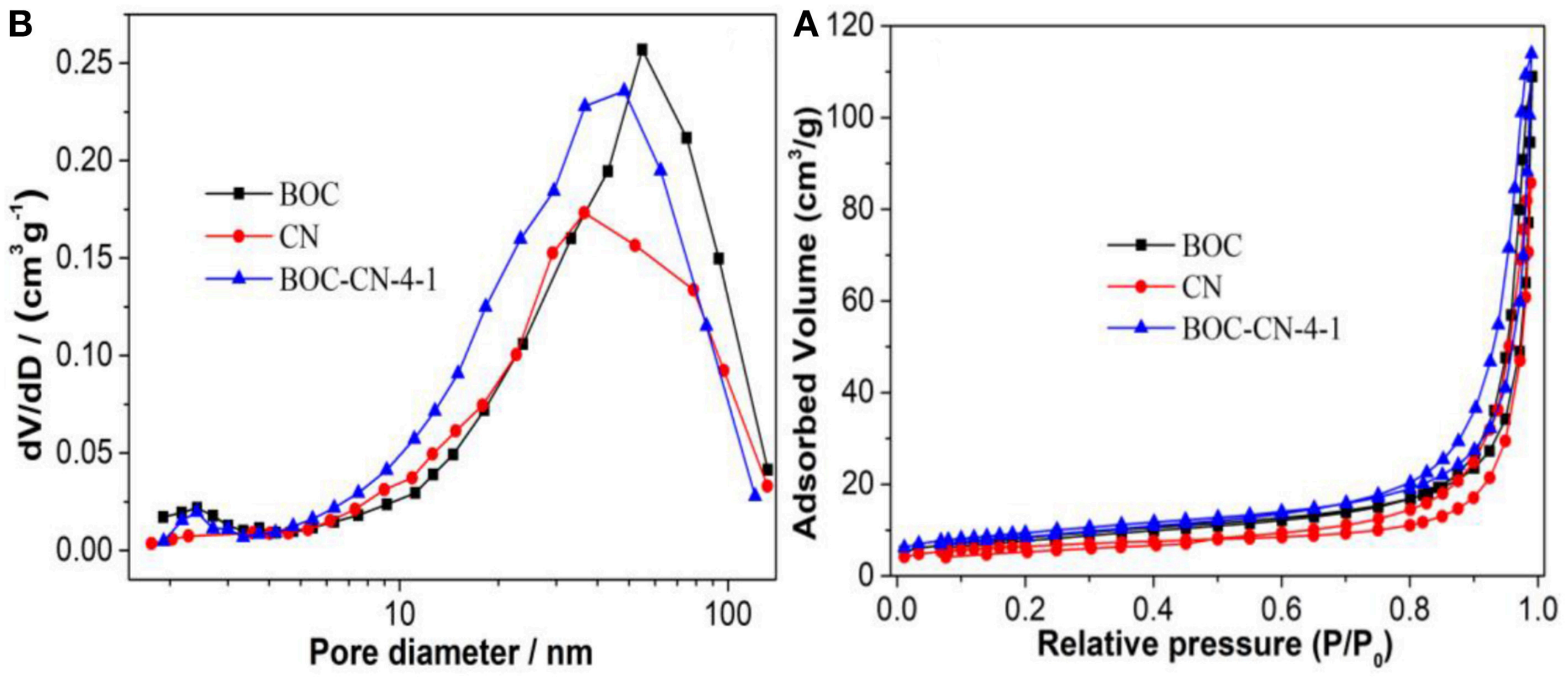
The adsorption-desorption isotherms **(A)** and pore size distribution curves **(B)** of BOC, CN, and BOC-CN-4-1 samples.

Figure 8A shows that the adsorption-desorption isotherms of all the samples are type IV according to the IUPAC classification, demonstrating that all the samples have mesopores (Dong et al., 2013; Zhang et al., 2014). However, the typical H3 hysteresis loop at low pressure can be ascribed to the aggregation of nanosheets with slit-like pores. Figure 8B further confirms the presence of mesopores in BOC, CN, and BOC-CN samples. In addition, the specific surface area (*S*_BET_) and total pore volume (*V*_p_) are 32.8 m^2^/g and 0.176 cm^3^/g for BOC-CN composites, which are higher than those of pure BOC (29.7 m^2^/g and 0.168 cm^3^/g) and CN (21.8 m^2^/g and 0.132 cm^3^/g), the enlarged *S*_BET_ and *V*_p_ of BOC-CN composites can be attributed to the stack of nanosheets layered by layered. The enlarged *S*_BET_ and *V*_p_ of BOC-CN composites can facilitate the reactants adsorb and transfer, and provide more active sites for the photocatalytic reaction.

**Figure 9:**
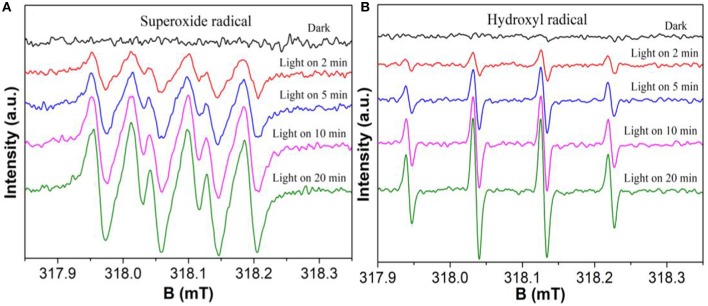
DMPO spin-trapping ESR spectra of BOC-CN-4-1 in methanol dispersion for DMPO-${•\text{O}}_{2}^{-}$ **(A)**, and in aqueous dispersion for DMPO-•OH **(B)**, respectively.

The electron spin resonance (ESR) experiments were further used to confirm the active species during the photocatalytic reaction process under visible light irradiation. As shown in Figures 9A,B, the superoxide (${•\text{O}}_{2}^{-}$) radicals and hydroxyl (•OH) radicals have been successfully detected by the ESR technique, respectively. Moreover, the intensity of all peaks increase significantly with the irradiation time, demonstrating that ${•\text{O}}_{2}^{-}$ and •OH are continuously generated during the reaction. The result shows that both ${•\text{O}}_{2}^{-}$ and •OH are the main photocatalytic reaction active species. The formation of ${•\text{O}}_{2}^{-}$ and •OH from the photochemical reaction shows in Equations 1–3 (Zhang et al., 2018a).

(1) $$\begin{array}{r} {\text{e}}^{-}+{\text{O}}_{2}\to •{\text{O}}_{2}^{-} \end{array}$$

(2) $$\begin{array}{r} •{\text{O}}_{2}^{-}+2{\text{H}}^{+}+{\text{e}}^{-}\to {\text{H}}_{2}{\text{O}}_{2} \end{array}$$

(3) $$\begin{array}{r} {\text{H}}_{2}{\text{O}}_{2}+{\text{e}}^{-}\to •\text{OH}+\text{O}{\text{H}}^{-} \end{array}$$

**Figure 10:**
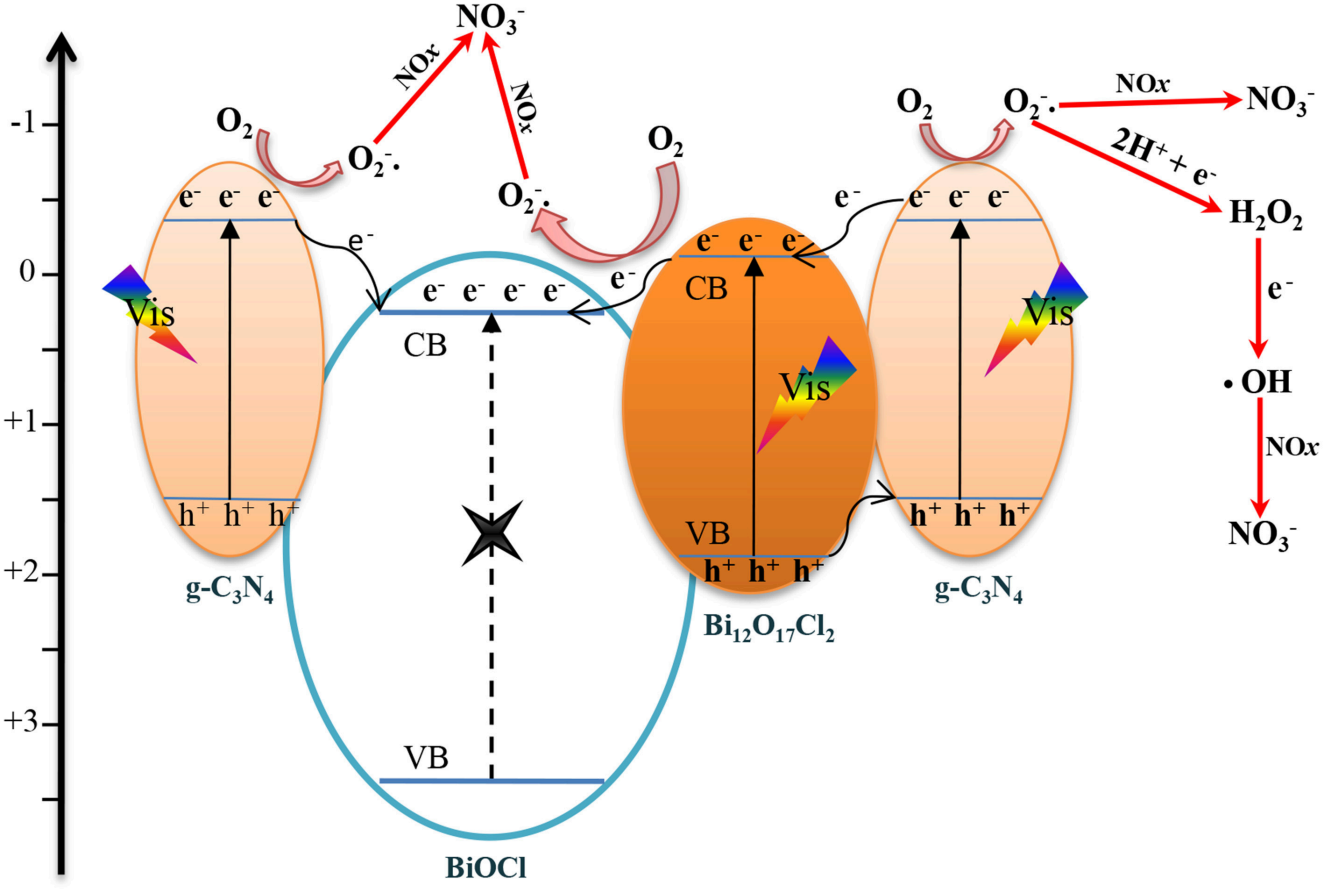
The proposed schematic mechanism for the photocatalytic reation at the BOC-CN heterojunction interface.

Furthermore, the possible mechanism for the photocatalytic reaction at the BOC-CN interface are presented in Figure 10. After the visible light irradiation, the Bi_12_O_17_Cl_2_ and g-C_3_N_4_ can be excited and then produce electron-hole pairs (Dong et al., 2013; Zhang et al., 2018b). On the one hand, the excited electrons in CB of g-C_3_N_4_ can directly transfer to CB of BiOCl (Zhang et al., 2013). On the other hand, the excited holes in VB of Bi_12_O_17_Cl_2_ can transfer to VB of g-C_3_N_4_, the excited electrons in CB of g-C_3_N_4_ can transfer to CB of Bi_12_O_17_Cl_2_, and the electrons can further transfer to the CB of BiOCl from the CB of Bi_12_O_17_Cl_2_, the band structures of the three components are well-matched resulting in efficient separation and transfer of the photo-induced carriers. Hence, the suitable band structures clearly show that the efficient electron-hole pair separation plays critical role in improving the photochemical reaction (Zhang et al., 2013, 2014).

**Figure 11:**
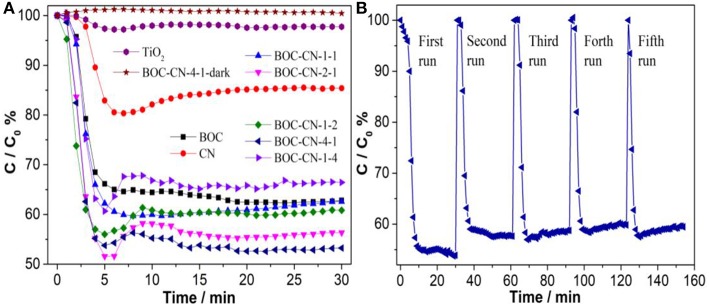
**(A)** Photocatalytic removal of NO over the as-obtained samples, **(B)** cycling runs of the BOC-CN-4-1 composite in air under visible light irradiation (λ > 420 nm).

The visible-light-induced photocatalytic activities of the TiO_2_, BOC, CN, and BOC-CN samples toward NO were revealed in Figure 11A. The pure BOC and CN only removed 36.2 and 14.6% of NO after 30 min visible light irradiation due to its fast recombination of photo-induced carriers, respectively. However, the visible-light-induced photocatalytic activities of the TiO_2_ sample can be neglected under the same conditions, indicating that the activity does not result from a UV-A light induced photocatalytic activity due to some trace of UV-A light after the cut-off filter. When the heterojunction was formed, the NO removal ratio over BOC-CN composites was increased to 46.8%. For ruling out and evidencing the NO degradation, the adsorption experiment of the optimized BOC-CN-4-1was carried out under dark condition, the result shows that adsorption property of NO over the BOC-CN-4-1-dark sample can also be ignored. The photocatalytic stability experiment of the BOC-CN-4-1 sample was evaluated by repeating the reaction for five runs under visible light irradiation. As shown in in Figure 11B, the photocatalytic performance shows slightly loss after five run, indicating that BOC-CN-4-1 photocatalyst possesses good photocatalytic stability. Interestingly, the BOC-CN composites exhibit even higher visible light photocatalytic activity than that of BiOBr/C_3_N_4_ (removal rate of 32.7%) and p-doped g-C_3_N_4_ (removal rate of 42.3%) (Sun et al., 2014; Zhang et al., 2016). The enhanced photocatalytic activity of BOC-CN can be attributed to the synergistic contribution of BOC and g-C_3_N_4_ with respect to the suitable band structure, enlarged *S*_BET_ and *V*_p_, improved visible light absorption, and efficient photo-induced carrier separation at the interface of BOC and g-C_3_N_4_ (Dong et al., 2013; Hou et al., 2013; Sun et al., 2014; Liu, 2015; Wei et al., 2016).

## Conclusion

In summary, we have synthesized ternary BOC-CN heterojunctions with outstanding visible light photocatalytic performance by self-assembly of BiOCl/Bi_12_O_17_Cl_2_ nanosheets on the surface of g-C_3_N_4_ nanosheets via a chemical deposition-precipitation method. The results reveal that g-C_3_N_4_, BiOCl, and Bi_12_O_17_Cl_2_ possess well-matched band structures, which is helpful to the separation and transport of photo-induced carriers. This work provides a new perspective for the design and fabrication of high performance and stable BiOCl/Bi_12_O_17_Cl_2_-based photocatalysts via a facile method at room temperature.

## Author Contributions

WZ: experiment, data analysis, and paper writing. YL: paper writing.

### Conflict of Interest Statement

The authors declare that the research was conducted in the absence of any commercial or financial relationships that could be construed as a potential conflict of interest.

## Supplementary Material

Supplementary Figure 1 The light spectra range of tungsten halogen lamp.
