# Supplementary figures and images for: Facile Synthesis of Ternary g-C3N4@BiOCl/Bi12O17Cl2 Composites With Excellent Visible Light Photocatalytic Activity for NO Removal
Source: Front Chem. 2019 Apr 11;7:231. doi: 10.3389/fchem.2019.00231 (PMC6470255; doi:10.3389/fchem.2019.00231)

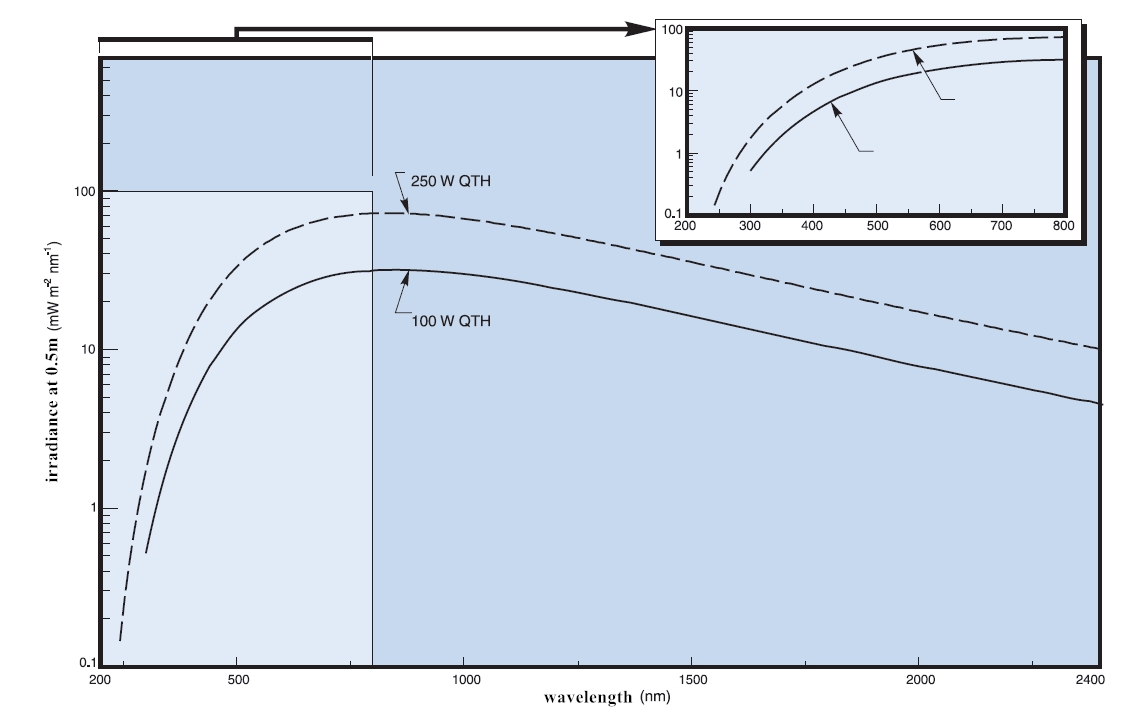

Supplement: Supplementary Figure 1 — The light spectra range of tungsten halogen lamp. [file Image_1.JPEG]
